# Flumazenil may improve gait and mentation in dogs presenting with marijuana toxicosis

**DOI:** 10.3389/fvets.2024.1516181

**Published:** 2024-12-18

**Authors:** Alyson H. Fitzgerald, Yuntao Zhang, Samuel Stewart, Scott A. Fritz, Alex M. Lynch, Monique Ramras, Stacy D. Meola

**Affiliations:** ^1^Department of Emergency and Critical Care, Wheat Ridge Animal Hospital, Wheat Ridge, CO, United States; ^2^Veterinary Diagnostic Lab, Kansas State University College of Veterinary Medicine, Manhattan, KS, United States; ^3^Ethos Veterinary Health, Woburn, MA, United States; ^4^Department of Emergency and Critical Care, North Carolina State University College of Veterinary Medicine, Raleigh, NC, United States; ^5^Department of Emergency and Critical Care, Cornell University College of Veterinary Medicine, Ithaca, NY, United States

**Keywords:** tetrahydrocannabidiol, ultraperformance liquid chromatography, toxicology, marijuana, flumazenil

## Abstract

**Introduction:**

Alongside the United States’ growing landscape of legalized recreational marijuana intended for humans, cases of canine marijuana toxicosis have been on the rise. Most commonly these dogs have mild clinical signs and respond well to supportive therapies. However, patients might still be ataxic, unable to walk, or remain heavily sedated at the time of discharge. Our hypothesis was that flumazenil would improve the level of consciousness, brainstem reflexes, gait, and stance in dogs with marijuana toxicosis.

**Methods:**

Seventeen dogs presenting for marijuana toxicosis were enrolled. MGCS and Canine Marijuana Severity Score (CMSS), were used to assess level of consciousness, brain stem reflexes, gait, and stance. Flumazenil 0.01 mg/kg was administered IV once. Baseline values immediately before flumazenil administration, 5 min, 15 min, and 30 min after flumazenil were recorded. Serum was collected and analyzed for delta-9-THC using ultraperformance liquid chromatography.

**Results:**

There was a significant change in MGCS and CMSS following flumazenil administration (*p* = 0.0033 and *p* ≤ 0.001). The median CMSS at baseline was 17 (10–19), at 5 min was 18 (10–21), at 15 min was 18 (12–22), and at 30 min was 19 (14–22). There was a significant difference between the concentration of delta-9-THC and clinical sign score (*p* = 0.0275).

**Discussion:**

The administration of flumazenil to dog affected by marijuana toxicosis might result in improved gait, stance, and level of consciousness. There might be some discriminative ability of the CMSS to stratify the severity level of canine marijuana toxicosis.

## Introduction

Over the past decade, marijuana intoxications have been more commonly seen in dogs across the United States (1–4). Recreational marijuana use has been legalized in several states, and access to this product has become more available to the public (1). Abnormal neurologic signs are seen in 99% of intoxicated dogs (5), characterized by depressed mentation, ataxia, mydriasis, and hyperesthesia. In addition, hypersalivation, bradycardia, emesis and urine dribbling are commonly reported (6). There can be considerable morbidity in affected cases, with mortality being reported in rare cases. Increased potency of marijuana products has been on the rise over the past several decades, which could be related to the morbidity associated with marijuana intoxication in dogs (7). Treatments are supportive in nature, but in more severely affected animals, treatments could include lipid emulsion or extracorporeal therapy (8).

The main psychoactive compound in marijuana is tetrahydrocannabinol (THC), which primarily acts on cannabinoid receptors 1 and 2 (CB1 and CB2) located in the brain and peripheral nervous system, respectively (6, 9, 10). Dogs have more cannabinoid receptors in their brain compared to humans, which could explain their increased sensitivity to marijuana products (9). The endocannabinoid system has a wide-reaching effect on numerous organ systems. Two well-known endocannabinoids, anandamide (ANA) and 2-arachidonoylglycerol (2-AG), regulate neurologic, immune, and inflammatory functions through their effect on CB1 and CB2 receptors. An *in vitro* study found that 2-AG might have a unique binding site with the *γ*-aminobutyric acid type A (GABA_A_) receptor (11, 12). Agonism of the GABA_A_ receptor is involved in sedation and inhibition of the arousal center which might be involved in sleep cycle and maintenance of anesthesia (13, 14). Flumazenil competitively antagonizes the benzodiazepine binding-site on GABA_A_ (15). In people, flumazenil is associated with reversing the effects of hypnotics, opioids, serotonin syndrome, alcohol intoxication, and other CNS depressants (14, 16–19). A case report described rapid improvement in mentation in two children that were comatose after ingesting marijuana (19). There is a knowledge gap as to whether flumazenil could be a useful adjunct in the management of THC intoxication in dogs.

The primary objective of this study was to evaluate the effect of flumazenil administration in dogs with marijuana toxicosis. We hypothesized that flumazenil administration would result in improved gait scores, brain stem reflexes, stance, and level of consciousness. A secondary objective was to evaluate a novel clinical scoring system in affected dogs, based on evaluation of gait, stance, brain stem reflexes, and level of consciousness.

## Materials and methods

Seventeen dogs presented to a private practice referral hospital with suspected or confirmed marijuana toxicosis, and were prospectively enrolled in this pilot study after obtaining client consent. Animal Care and Use Committee approval was obtained prior to initiating the study. Two scoring systems were used simultaneously: the Modified Glasgow Coma Score (MGCS) and a novel marijuana toxicosis scoring system titled the Canine Marijuana Severity Score (CMSS), which was adapted from both the MGCS and the Scale for the Assessment and Rating of Ataxia (Supplementary Table S1) (20, 21). Veterinary technicians and veterinarians were trained on how to use both scoring systems. Technicians, associate veterinarians, and senior emergency veterinarians performed the scoring.

Of the 17 dogs enrolled, 5/17 (29.4%) were toy breeds [(Chihuahua (2), Maltese mix (1), Terrier mix (1), Yorkie mix (1)]. Other breeds represented include the Pembroke Welsh Corgi (1), Australian Cattle Dog mix (1), Newfoundland (1), Goldendoodle (1), Lab mix (1), Pug (1), Cocker spaniel mix (1), Mini Australian Shepherd (1), Boxer mix (1), Shiba Inu (1), Saint Bernard (1) and Labrador Retriever (1). There were 10 males, (8 castrated, 2 intact) and 7 females (6 spayed, 1 intact). About 30% (5/17) were less than 1 year of age with a median age of 3 years (range: 5 months to 12 years). The median weight was 10.5 kg (2.9–58 kg).

**Figure 1:**
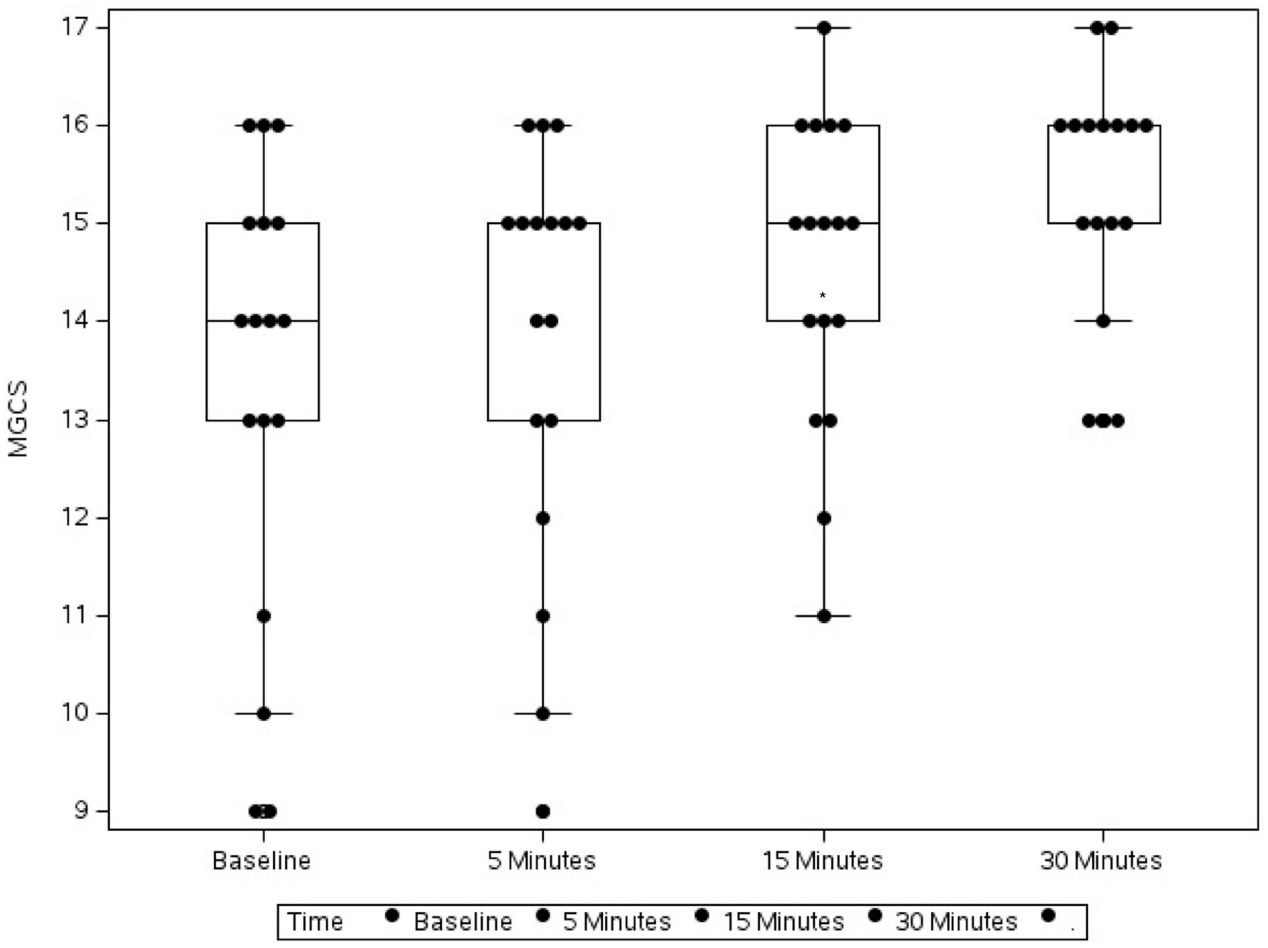
Box plot showing the change in Modified Glasgow Coma Scale (MGCS) in 17 dogs following flumazenil administration at baseline (median 16, range 9–17), 5 min (median 16, range 12–17), 15 min (median 16, range 13–16), and 30 min (median 17, range 13–18) * Indicates statistical significance.

**Figure 2:**
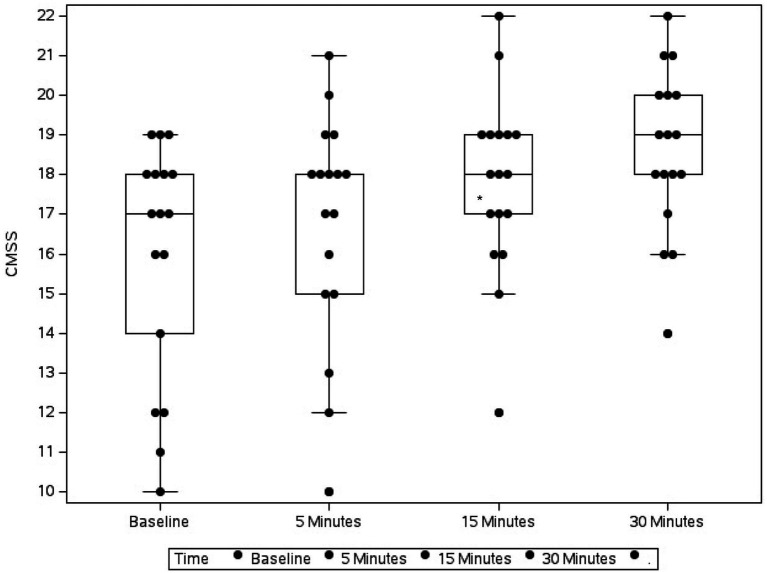
Box plot showing the change in Canine Marijuana Severity Score (CMSS) in 17 dogs following flumazenil administration at baseline (median 17, range 10–19), 5 min (median 18, range 10–21), 15 min (median 18, range 12–22), and 30 min (median 19, range 14–22) * Indicates statistical significance.

Baseline assessment scores were recorded at presentation, and then 3 mL of whole blood was collected via jugular or cephalic venipuncture and immediately placed in a plastic tube without any additive. A single intravenous injection of flumazenil (0.01 mg/kg) was administered over approximately 1 min. The MGCS and CMSS were re-assessed 5, 15, and 30 min after flumazenil administration (Figures 1, 2). In addition, heart rate, urine dribbling, hyperesthesia, vocalization, and vomiting were recorded during the monitoring period. After the 30-min time point, all subsequent treatment interventions provided to clinical cases were at the discretion of the primary clinician.

The collected blood was immediately centrifuged at 3,400 rpm for 10 min. The serum was then carefully pipetted into separate plastic cryovials and frozen at −20°C or −80°C. Samples collected over the first 2-months were stored in a −20°C freezer. After that time point, the hospital obtained a −80°C freezer and samples were subsequently stored there. After 12 months of sample collection, samples were shipped together overnight on ice to for batch analysis of THC via ultra-performance liquid chromatography electrospray ionization and mass spectrometry (UPLC-ESI-MS/MS) using a previously validated method for canine serum (22).

## Statistical methods

Data were analyzed using statistical software (GraphPad Prism 10.1.2). Continuous variables were assessed for normality using Q-Q plots and Shapiro–Wilk tests. Normality was not met for any of the variables being evaluated, including following log transformations. Change in MGCS and CMSS from baseline to 30 min following flumazenil administration were analyzed using Friedman tests. The change in median CMSS was analyzed between several time points using Dunn’s multiple comparison tests. For analysis, serum THC concentrations were grouped into 0–100 ng/mL, 100–200 ng/mL and >200 ng/mL and were compared to baseline CMSS using a Kruskal-Wallis test. Comparison of patient weights between each of the THC range categories was also calculated using a Kruskal-Wallis test. Significance was determined by a *p*-value < 0.05.

## Results

Of the 17 patients enrolled, 4/17 had confirmed marijuana exposure (2 chocolate-based marijuana products, 1 marijuana plant material and 1 gummy product), and 13/17 were suspected exposures based on the history and the patients’ physical examination. The most common clinical signs were ataxia, hyperesthesia, urine dribbling and cranial nerve deficits (Table 1). Heart rates were recorded in 10/17 patients and rectal temperatures were recorded in 15/17 patients. Patients were more often hyperthermic (rectal temperature ≥ 102.5, 6/15) and tachycardic (heart rate > 120 beats per minute) in this group. Only 2/17 (11.8%) patients were treated as inpatients and all others were treated as outpatients. One patient had a history of well-controlled seizures and received phenobarbital and cannabidiol supplements. No other pertinent medical history was noted for any other patient.

**Table 1:** Clinical sign and physical exam finding frequency.

Clinical sign or physical exam finding	Frequency
Ataxia	12/17 (71%)
Hyperesthesia	11/17 (65%)
Urine dribbling	9/17 (53%)
Cranial nerve deficits	10/17 (59%)
Delayed PLR	10/17 (59%)
Absent or reduced menace response	3/17 (18%)
Absent conscious proprioception	1/17 (6%)
Decreased palpebral response	1/17 (6%)
Strabismus	1/17 (6%)
Decreased nasal sensation	1/17 (6%)
Hyperthermia	6/15 (40%)
Hypothermia	1/15 (7%)
Tachycardia	4/10 (40%)
Bradycardia	3/10 (30%)

Over the 30-min monitoring period there was a significant change in median baseline scores following flumazenil administration for both MGCS and CMSS (Figures 1, 2). The baseline median MGCS was 16 (9–17), at 5 min post-flumazenil was 16 (12–17), at 15 min was 16 (13–16) and at 30 min was 17 (13–18). The median CMSS at baseline was 17 (10–19), at 5 min was 18 (10–21), at 15 min was 18 (12–22), and at 30 min was 19 (14–22). Following Dunn’s multiple comparisons there was a statistically significant improvement in MGCS and CMSS scores from 0 to 30 min (Table 2).

**Table 2:** Rank sum difference in CMSS.

Comparison	Mean difference	Adjusted *p*-value^a^
0 min vs. 5 min	+11.0	0.86
0 min vs. 15 min	+19.5	0.06
0 min vs. 30 min	+29.5	**0.0005**
5 min vs. 15 min	+8.5	>0.99
5 min vs. 30 min	+18.5	0.0839
15 min vs. 30 min	+10.0	>0.99

a *p-value calculated using Dunn’s multiple comparison test, n = 17. Bolded value indicates statistical significance.*

Serum THC was analyzed in all 17 patients. Detectable concentrations of THC were identified in 16 of 17 dogs (94%). The average serum THC concentration in this cohort was 214.93 ng/mL (range: 0–1725.4 ng/mL). There was a significant inverse relationship between the serum concentration of THC and baseline CMSS (Table 3). In patients with serum THC concentrations between 0 and 100 ng/mL the average score was 18, between 100 and 200 ng/mL the average score was 16.5, and >200 ng/mL the average score was 11.5. There were no significant differences when comparing patient weight to each of the THC concentration range categories (*p* = 0.19). In 4/17 patients, there was no change from baseline CMSS to 30 min after flumazenil administration. Additionally, 1/4 dogs with no change in CMSS following flumazenil also had a decreased menace response in the right eye and absent nasal sensation bilaterally on physical exam. This prompted additional neurologic work up which resulted in the diagnosis of meningoencephalitis of unknown etiology based on MRI and THC toxicosis based on the presence of THC in serum. This patient had a normal CSF tap and diffuse meningeal enhancement on MRI. One patient vomited and and two patients vocalized during the monitoring period. Aside from one patient vomiting, no other adverse effects were recorded. The patient with a previous history of seizures had no reported seizure activity 24 h after discharge.

**Table 3:** Comparison of baseline CMSS and serum THC concentration.

[Δ9-THC] (ng/mL)	0–100	100–200	>200
Median	18.0	16.5	11.5
Range	14.0–19.0	10.0–18.0	11.0–12.0
*p*-value^a^	0.0275		

a *p-value calculated using Kruskal-Wallis test, n = 17.*

## Discussion

We evaluated the impact of flumazenil administration as an adjunctive treatment for dogs with symptomatic marijuana toxicosis. Symptoms were assessed using an established scoring scheme (MGCS) and a novel score (CMSS). Flumazenil administration was associated with improvements in both scores within 30-min, consistent with reduced severity of neurologic signs. Quantifiable THC concentrations were identified in all but one dog. Dogs with lower CMSS tended to have significantly higher serum THC concentrations. This novel score therefore shows promise as a novel indicator of clinical severity in affected dogs.

Marijuana toxicosis in dogs is common, usually causing significant but self-limiting morbidity in dogs. Fatalities have been reported however and more specific interventions might be necessary to hasten clinical recovery (1). In veterinary medicine, flumazenil is primarily used to reverse paradoxical excitation and prolonged recovery times following benzodiazepine administration (23). Flumazenil is used in human medicine to reverse the effects of several toxicants, including marijuana (14, 16–19). The aim of this study, therefore, was to assess whether flumazenil administration was associated with improvement in clinical signs in dogs with symptomatic THC toxicosis. In our study, 13/17 and 10/17 affected dogs had improved ( ≥ 1 change in score from baseline) CMSS and MGCS scores, respectively, within 30-min of flumazenil administration, consistent with less severe neurologic signs (Supplementary Tables S2, S3). MGCS may be a less sensitive scoring system compared to the novel CMSS. The frequency of ataxia in this cohort was 70% and the MGCS does not assess this parameter unlike the CMSS. We did not observe statistical improvement in either score within 5 or 15-min of flumazenil administration. Interestingly, the peak effect of flumazenil occurs 6–10 min after administration in people (15), with an elimination half-life of 40–60 min. In dogs given tiletamine-zolazepam, there is a dose-dependent improvement in the ability to stand and walk 10 min after flumazenil administration (23). It is possible that an additional or higher dose of flumazenil would have achieved a faster clinical response. It is also plausible that the weak GABA_A_ antagonist effect of flumazenil on THC is delayed in comparison to the stronger competitive antagonistic effect seen with flumazenil and benzodiazepines. It is possible that the dogs included in our study might have improved on their own despite the use of flumazenil. However, given the biological half-life of THC is approximately 30 h, spontaneous clinically overt improvements within 30 min are less plausible (6, 9).

Cranial nerve deficits were present in 58% of affected dogs in our study, which appears higher than previous reports in the veterinary literature (4, 8). Delayed PLR in one or both eyes was the most common cranial nerve deficit noted in this cohort. Binagia et al. recently published a large retrospective study of patients with canine marijuana toxicosis, reporting delayed or absent PLR in 1.3% of cases (4). The reason for these differences is not known, although interobserver variation between clinicians performing cranial nerve exams is plausible. The PLR was assessed as normal by the end of the 30-min monitoring period in 6/10 patients suggesting there was an observable change in speed in the PLR response. In humans there appears to be a correlation between higher THC concentrations and more delayed PLR reflexes. The average serum THC concentration in this cohort of dogs was higher than previously reported, which could explain the increased frequency of PLR abnormalities (22).

One dog in this pilot study was diagnosed with both marijuana toxicosis and meningitis of unknown etiology. In humans, there is a possible association between THC and the acute onset of necrotizing encephalitis without evidence of infectious etiology (24). In dogs with steroid-responsive meningitis-arteritis there are increased circulating endocannabinoids, and THC can directly increase the concentration of plasma endocannabinoids (10, 25). While there is literature showing the anti-inflammatory properties of endocannabinoids, both *in vivo* and *in vitro* work has shown that AG-2 can play a pro-inflammatory role via the production of reactive oxygen species, promoting white blood cell migration and stimulating pro-inflammatory cytokine release (10). This suggests that the immunomodulatory action of THC could contribute to a pro-inflammatory state that led to the onset of meningoencephalitis in this patient.

There are sparse reports in the human literature stating reversal with flumazenil can cause seizures and cardiac arrhythmias. Reports are often associated with high flumazenil dosing, history of chronic benzodiazepine use, or history of multiple drug co-ingestion (15). In a large observational study in humans, flumazenil administration did not result in seizures or arrhythmias in those patients despite seizure history and multiple drug co-ingestion (18). Other minor side effects of flumazenil in humans are vomiting, salivation, and anxiety (18). In a study where beagles are given flumazenil to reverse tiletamine-zolazepam, seizures, hypersalivation, vocalization, and opisthotonos were reported at doses 8–16 times the dose used in this pilot study (0.01 mg/kg) (23). One dog in this pilot study vomited during the monitoring period but had a history of vomiting prior to presentation, which could be related to prior marijuana ingestion or flumazenil administration. One dog enrolled had a history of well-controlled seizures on phenobarbital and CBD and was seizure-free for more than a year, and no seizure activity was noted during monitoring or in the 24 h after discharge after follow-up with the owner. However, given the small cohort in this study it cannot be determined whether there is an increased risk of seizures when giving flumazenil at standard doses to patients with a history of seizures. It also is prudent to mention that if a dog does have a seizure after flumazenil administration, benzodiazepines may be less effective in terminating the seizure.

In our study, dogs with higher serum THC concentrations had lower CMSS at baseline. This could indicate that these patients were more severely affected; however, we collected serum on presentation and dogs were presented at different time points post-exposure. For three of these dogs, serum was stored in a −20°C freezer until a −80°C freezer was later purchased by the hospital. Resin-based marijuana products with varying levels of THC concentrations showed no statistical difference in THC concentrations over a four-year time period when stored at −20°C (26). Therefore, differences in THC stability between the samples stored in −20°C and −80°C freezer are unlikely.

One patient had a serum THC concentration > 1,500 ng/mL, the highest in this cohort, and concurrently had the lowest CMSS score (11/22). There were only 3 patients with CMSS scores of 12 or less. Those patients, while all having a significant change from baseline CMSS (+2 or greater) across the 30 min, 2/3 had dramatic changes in CMSS from baseline (+5 and + 6). Two of the dogs’ gait were unable to be assessed to due severe impairment (gait contributed 0 to their score) which impacted their overall baseline score. All patients had detectable concentrations of THC in serum except for one patient whose score was unchanged throughout the monitoring period. This patient’s serum was stored at −80°C immediately after collection until shipment for analysis.

Limitations of this study include lack of a control group, small sample size, different scoring assessors, different storage conditions, and the paucity of moderate and severe marijuana toxicosis patients. There was a significant change in CMSS and MGCS after 30 min following flumazenil administration and for some patients this resulted in either mild clinical improvement or no change from baseline. While it appears that flumazenil is well-tolerated in this small cohort of patients, it should still be used with caution depending on the patient factors and exposure scenario.

In conclusion, flumazenil might be considered as an adjunctive treatment for marijuana toxicosis based on our observations of improved neurological symptoms within 30 min of administration in affected dogs. The CMSS scoring system appeared to identify dogs with higher serum THC concentrations and more severe symptoms. Finally, we report on a novel case of concurrent marijuana toxicosis and meningitis of unknown etiology in one dog.

## Data Availability

The raw data supporting the conclusions of this article will be made available by the authors, without undue reservation.
