# Supplementary material for: Flumazenil may improve gait and mentation in dogs presenting with marijuana toxicosis
Source: Front Vet Sci. 2024 Dec 18;11:1516181. doi: 10.3389/fvets.2024.1516181 (PMC11688805; doi:10.3389/fvets.2024.1516181)
Supplement: Supplementary file 1 [file Table_1.DOCX]

Supplemental Table 1: Canine Marijuana Severity Score (CMSS)

| Brainstem reflexes | (6) normal PLR and oculocephalic reflexes | (5) slow PLR and normal to reduced oculocephalic reflexes | (4) bilateral unresponsive miosis with normal to reduced oculocephalic reflexes | (3) pinpoint pupils with reduced to absent oculocephalic reflexes | (2) unilateral, unresponsive mydriasis with reduced to absent oculocephalic reflexes | (1) bilateral unresponsive mydriasis with reduced to absent oculocephalic reflexes |
| --- | --- | --- | --- | --- | --- | --- |
| Level of consciousness | (6) occasional period of alertness and responsive to environment | (5) depression or delirium, capable of responding, but response may be inappropriate | (4) semicomatose, responsive to visual stimuli | (3) semicomatose, responsive only to auditory stimuli | (2) semicomatose, responsive only to repeated noxious stimuli | (1) comatose, unresponsive to repeated noxious stimuli |
| Gait | (5) normal, no difficulties in walking or turning (up to one misstep allowed) | (4) slight difficulties, only visible when walking 10 consecutive steps | (3) considerable staggering, difficulties in turning without support | (2) marked staggering, intermittent support required | (1) severe staggering, permanent support required |  |
| Stance | (5) normal, able to stand without sway for >10s | (4) able to stand without sway, but for <10s | (3) able to stand for >10s with sway and/or with intermittent support | (2) able to stand for >10s with constant support | (1) unable to stand for >10s even with constant support |  |
