# Supplementary material for: Flumazenil may improve gait and mentation in dogs presenting with marijuana toxicosis
Source: Front Vet Sci. 2024 Dec 18;11:1516181. doi: 10.3389/fvets.2024.1516181 (PMC11688805; doi:10.3389/fvets.2024.1516181)
Supplement: Supplementary file 2 [file Table_2.DOCX]

Supplemental Table 2: Individual CMSS scores over time following flumazenil administration

| Patient | Time (minutes) | Brainstem reflexes | Level of consciousness | Gait | Stance | Total Scores (out of 22) |
| --- | --- | --- | --- | --- | --- | --- |
| Dog 1 | 0 | 5 | 5 | 5 | 3 | 18 |
|  | 5 | 5 | 5 | 5 | 3 | 18 |
|  | 15 | 6 | 6 | 5 | 5 | 22 |
|  | 30 | 6 | 6 | 5 | 5 | 22 |
| Dog 2 | 0 | 5 | 6 | 5 | 3 | 19 |
|  | 5 | 5 | 6 | 5 | 3 | 19 |
|  | 15 | 5 | 6 | 5 | 5 | 21 |
|  | 30 | 5 | 6 | 5 | 5 | 21 |
| Dog 3 | 0 | 6 | 5 | 3 | 3 | 17 |
|  | 5 | 6 | 6 | 3 | 3 | 18 |
|  | 15 | 6 | 6 | 4 | 3 | 19 |
|  | 30 | 6 | 6 | 4 | 3 | 19 |
| Dog 4 | 0 | 5 | 6 | 4 | 3 | 18 |
|  | 5 | 6 | 6 | 4 | 4 | 20 |
|  | 15 | 6 | 5 | 4 | 4 | 19 |
|  | 30 | 6 | 5 | 5 | 4 | 20 |
| Dog 5 | 0 | 6 | 3 | N/A | 3 | 12 |
|  | 5 | 6 | 5 | 3 | 3 | 17 |
|  | 15 | 6 | 5 | 3 | 3 | 17 |
|  | 30 | 6 | 6 | 4 | 4 | 20 |
| Dog 6 | 0 | 6 | 6 | 2 | 3 | 17 |
|  | 5 | 6 | 6 | N/A | 3 | 15 |
|  | 15 | 6 | 6 | 3 | 3 | 18 |
|  | 30 | 6 | 6 | 5 | 3 | 20 |
| Dog 7 | 0 | 6 | 5 | 3 | 3 | 17 |
|  | 5 | 6 | 6 | 3 | 3 | 18 |
|  | 15 | 6 | 6 | 4 | 3 | 19 |
|  | 30 | 6 | 6 | 4 | 3 | 19 |
| Dog 8 | 0 | 5 | 5 | 3 | 3 | 16 |
|  | 5 | 5 | 5 | 4 | 3 | 17 |
|  | 15 | 5 | 5 | 3 | 3 | 16 |
|  | 30 | 5 | 5 | 3 | 3 | 16 |
| Dog 9 | 0 | 6 | 5 | 2 | 1 | 14 |
|  | 5 | 6 | 5 | 2 | 2 | 15 |
|  | 15 | 6 | 5 | 2 | 2 | 15 |
|  | 30 | 6 | 5 | 4 | 3 | 18 |
| Dog 10 | 0 | 5 | 5 | 3 | 3 | 16 |
|  | 5 | 5 | 5 | 3 | 3 | 16 |
|  | 15 | 5 | 5 | 4 | 3 | 17 |
|  | 30 | 5 | 5 | 4 | 3 | 17 |
| Dog 11 | 0 | 5 | 6 | 4 | 3 | 18 |
|  | 5 | 5 | 6 | 4 | 3 | 18 |
|  | 15 | 5 | 6 | 4 | 3 | 18 |
|  | 30 | 5 | 6 | 4 | 3 | 18 |
| Dog 12 | 0 | 5 | 4 | 1 | 1 | 11 |
|  | 5 | 5 | 4 | 1 | 3 | 13 |
|  | 15 | 5 | 4 | 3 | 4 | 16 |
|  | 30 | 5 | 5 | 3 | 3 | 16 |
| Dog 13 | 0 | 6 | 3 | N/A | 1 | 10 |
|  | 5 | 6 | 3 | N/A | 1 | 10 |
|  | 15 | 6 | 5 | 3 | 3 | 17 |
|  | 30 | 6 | 5 | 4 | 3 | 18 |
| Dog 14 | 0 | 5 | 5 | 4 | 4 | 18 |
|  | 5 | 5 | 6 | 4 | 4 | 19 |
|  | 15 | 5 | 6 | 4 | 4 | 19 |
|  | 30 | 5 | 6 | 5 | 5 | 21 |
| Dog 15 | 0 | 5 | 6 | 5 | 3 | 19 |
|  | 5 | 5 | 6 | 5 | 5 | 21 |
|  | 15 | 6 | 6 | 4 | 3 | 19 |
|  | 30 | 6 | 6 | 4 | 3 | 19 |
| Dog 16 | 0 | 5 | 5 | 1 | 1 | 12 |
|  | 5 | 5 | 5 | 1 | 1 | 12 |
|  | 15 | 5 | 5 | 1 | 1 | 12 |
|  | 30 | 6 | 6 | 1 | 1 | 14 |
| Dog 17 | 0 | 6 | 6 | 4 | 3 | 19 |
|  | 5 | 6 | 5 | 4 | 3 | 18 |
|  | 15 | 6 | 5 | 4 | 3 | 18 |
|  | 30 | 6 | 5 | 4 | 3 | 18 |
